# cGAS guards against chromosome end-to-end fusions during mitosis and facilitates replicative senescence

**DOI:** 10.1007/s13238-021-00879-y

**Published:** 2021-10-22

**Authors:** Xiaocui Li, Xiaojuan Li, Chen Xie, Sihui Cai, Mengqiu Li, Heping Jin, Shu Wu, Jun Cui, Haiying Liu, Yong Zhao

**Affiliations:** 1 MOE Key Laboratory of Gene Function and Regulation, State Key Laboratory of Biocontrol, School of Life Sciences, Sun Yat-sen University, Guangzhou, 510006 China; 2 State Key Laboratory of Oncology in South China, Collaborative Innovation Center for Cancer Medicine, Sun Yat-sen University Cancer Center, Guangzhou, 510006 China; 3 Department of Biochemistry, School of Life Sciences, Sun Yat-sen University, Guangzhou, 510006 China

**Keywords:** cGAS, telomeres, chromosome end-to-end fusion, DNA damage response, non-homologous end joining, mitosis, genome stability

## Abstract

As a sensor of cytosolic DNA, the role of cyclic GMP-AMP synthase (cGAS) in innate immune response is well established, yet how its functions in different biological conditions remain to be elucidated. Here, we identify cGAS as an essential regulator in inhibiting mitotic DNA double-strand break (DSB) repair and protecting short telomeres from end-to-end fusion independent of the canonical cGAS-STING pathway. cGAS associates with telomeric/subtelomeric DNA during mitosis when TRF1/TRF2/POT1 are deficient on telomeres. Depletion of cGAS leads to mitotic chromosome end-to-end fusions predominantly occurring between short telomeres. Mechanistically, cGAS interacts with CDK1 and positions them to chromosome ends. Thus, CDK1 inhibits mitotic non-homologous end joining (NHEJ) by blocking the recruitment of RNF8. cGAS-deficient human primary cells are defective in entering replicative senescence and display chromosome end-to-end fusions, genome instability and prolonged growth arrest. Altogether, cGAS safeguards genome stability by controlling mitotic DSB repair to inhibit mitotic chromosome end-to-end fusions, thus facilitating replicative senescence.

**Supplementary Information:**

The online version contains supplementary material available at 10.1007/s13238-021-00879-y.

## INTRODUCTION

The cyclic GMP-AMP synthase (cGAS) is widely regarded as an innate immune sensor that detects the presence of DNA in cytosol, including microbial and self-DNA (Sun et al., 2013; Hartlova et al., 2015; West et al., 2015). Upon recognition of double-stranded DNA (dsDNA), cGAS catalyzes the synthesis of the second messenger cyclic GMP-AMP (cGAMP) (Wu et al., 2013). cGAMP binds and activates STING (Stimulator of Interferon Genes) on the endoplasmic reticulum (ER) surface (Ishikawa and Barber, 2008). STING then traffics from the ER to the Golgi apparatus and activates transcription factors IRF3 and NF-κB via kinases TBK1 and IKKα/β, respectively (Tanaka and Chen, 2012). Subsequently, IRF3 and NF-κB enter the nucleus to upregulate the production of interferons (IFNs) and other cytokines. The cGAS-STING pathway plays an important role in innate immunity and viral defense. Dysregulation of the cGAS-STING pathway has been related to many disorders including infections, inflammatory diseases, neurodegeneration, cancers and aging-associated diseases. Thus the cGAS-STING pathway must be properly regulated. Recently, more and more specific small-molecule agonists and antagonists of the cGAS-STING pathway were developed (Ding et al., 2020; Decout et al., 2021; Fryer et al., 2021). They are not only very useful as research tools but also beneficial as potential therapeutic agents for the treatment of a variety of human diseases, such as aicardi-goutieres syndrome (AGS) and amyotrophic lateral sclerosis (ALS) (Dai et al., 2019; Yu et al., 2020).

Although cGAS is best known as a cytosolic protein, it binds to chromosomes following mitotic nuclear envelope rupture (Yang et al., 2017; Zierhut et al., 2019). It has been reported that nuclear cGAS are enriched on multiple loci of mitotic chromosome (Gentili et al., 2019). This phenomenon raises the question of what is the role of cGAS on chromosome during mitosis since cGAS’s canonical function in innate immune response is deactivated during mitosis (Zierhut et al., 2019; Boyer et al., 2020; Kujirai et al., 2020; Michalski et al., 2020; Pathare et al., 2020; Zhao et al., 2020; Li et al., 2021). In addition, the fact that STING is often silent while cGAS is rarely missing further implies that cGAS has STING-independent function that may be essential in cells. Up to now, whether and how cGAS functions in other cellular processes remain drawing hot attention. Recently, cGAS is reported to contribute to the regulation of homologous recombination and DNA replication in the nuclear genome during S/G_2_ phase (Liu et al., 2018; Jiang et al., 2019; Chen. et al., 2020), which is independent of the cGAS-STING axis. The function of cGAS during mitosis is not fully understood.

In human cells, telomeres are composed of TTAGGG repeats and associated proteins termed “shelterin” (de Lange, 2005). Telomeres prevent the natural end of chromosomes from being recognized as DNA double-stranded break (DSB), which might otherwise activate DNA damage response (DDR) leading to DNA degradation, end-to-end fusion and illegitimate recombination (d'Adda di Fagagna et al., 2003). It is reported that TRF2, a key component of shelterin, are removed from telomeres during mitosis (Cesare et al., 2009; Hayashi et al., 2012). In consistent with this, metaphase-TIF (Telomere Dysfunction Induced Foci) are the most predominant form of spontaneous DDR in mitotic cells (Nakamura et al., 2008; Cesare et al., 2009; Thanasoula et al., 2010; Kaul et al., 2011). Despite of activation of DDR, detrimental DNA-repair and resulting telomere fusion do not occur during mitosis. It is thus conceivable that mitotic DNA repair is inhibited on telomeres by the mechanism distinct from TRF2. So far, the most likely mechanism is that CDK1 phosphorylates RNF8 and 53BP1 to prevent their recruitment to DNA damage sites during mitosis (Orthwein et al., 2014). However, how CDK1 executes its function on telomeres is unknown.

In this report, we observed that cGAS occupies TRF2-deficient telomeres in mitosis and that depletion of cGAS leads to mitotic chromosome end-to-end fusion. Interestingly, we revealed that DDR is activated on short telomeres during mitosis, but blocked at the step of MDC1, which is supposed to be followed by recruitment of RNF8. Our mechanistic study discovered that CDK1 is positioned to chromosome ends by interacting with NTase domain of cGAS, wherein CDK1 disrupts the interaction between MDC1 and RNF8 via phosphorylation of RNF8. This uncanonical function of cGAS helps explain the paradox that short telomere activates DDR without inducing chromosome end-to-end fusion. More importantly, inhibition of telomere fusion by cGAS enables the activation of replicative senescence. If not, the fusion would quench DDR on critically short telomeres and allow cells to bypass senescence, thus providing a potential for transformation.

## RESULTS

### cGAS occupies shelterin-deficient telomeres during mitosis

**Figure 1:**
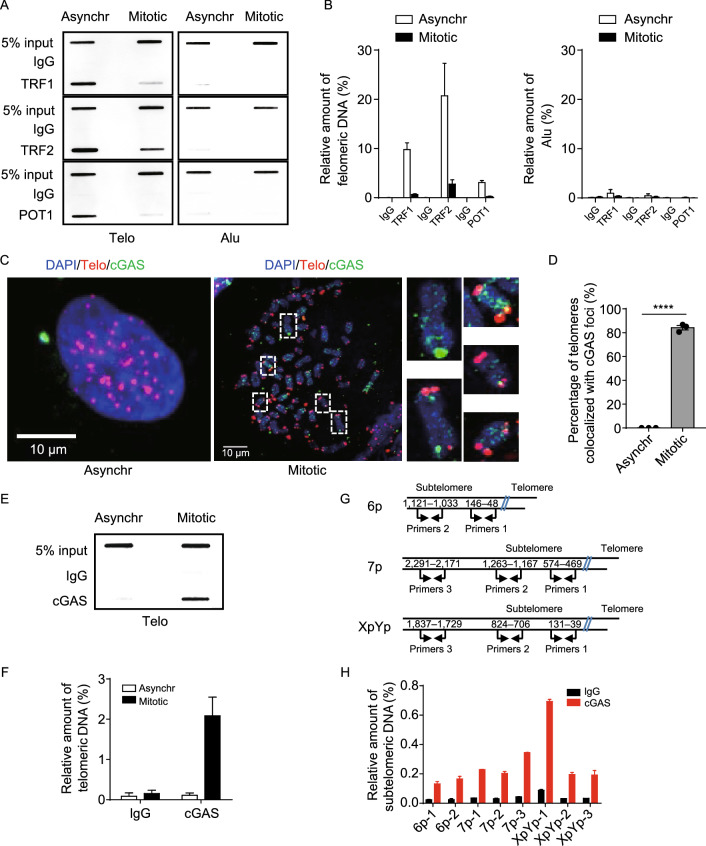
**cGAS binds to telomeres and subtelomeres during mitosis**. (A) ChIP analysis of TRF1, TRF2 and POT1 associating with telomeres in asynchronous (Asynchr) or mitotic U2OS cells. Cells were either asynchronous or synchronized at G_1_/S, released for 8 h and then treated with colcemid for 12 h. ChIP enriched DNA was used for slot blot and hybridization using telomeric G-rich probe or Alu probe. (B) Quantification of (A). The relative amount of enriched telomeric DNA was calculated (ChIP/Input, %). Alu was used as a control. All values are the average ± SEM of three independent experiments. (C) Visualization of endogenous cGAS and telomeres in asynchronous (Asynchr) or mitotic U2OS cells. Cells were treated as in (A) and then subjected to metaphase spread followed by IF/FISH. Scale bars, 10 μm. (D) Quantification of (C). Percentage of telomeres colocalizing with cGAS foci was calculated. All values are the average ± SEM of three independent experiments. (E) ChIP analysis of cGAS associating with telomeres in asynchronous (Asynchr) or mitotic U2OS cells. (F) Quantification of (E). The relative amount of enriched telomeric DNA was calculated (ChIP Input, %). All values are the average ± SEM of three independent experiments. (G) Schematic diagram showing specific primers for determining subtelomeric DNA of 6p, 7p or XpYp chromosomes. (H) cGAS-ChIP coupled with q-PCR to detect subtelomeric DNA using primers in (G). The relative amount of enriched subtelomeric DNA was calculated (ChIP/Input, %). All values are the average ± SEM of three independent experiments. Student’s unpaired two-tailed *t*-test was used to determine the statistical significance (****P < 0.0001). See also Fig. S1

To investigate end protection of telomeres by shelterin during mitosis, U2OS and HeLa cells were synchronized in mitosis and assayed to determine the occupancy of TRF1, TRF2 and POT1 at telomeres. Our chromatin immunoprecipitation (ChIP) data showed that numbers of telomere-associated TRF1, TRF2 and POT1 decline by 90%, 85% and 90%, respectively, during mitosis, as compared to asynchronized cells (Figs. 1A, 1B, S1A and S1B), whereas protein levels of TRF1, TRF2 and POT1 remain unchanged (Fig. S1C). These results are consistent with previous finding that TRF2 is absent on telomeres in prolonged mitotic arrest human cells and indicate that telomeres face the risk of deprotection during mitosis (Hayashi et al., 2012).

It has been reported previously that nuclear membrane dissolution during mitosis allows cGAS to associate with centromeric satellite DNA (Gentili et al., 2019). Here, using metaphase chromosome spreads coupled with co-staining of cGAS (ImmunoFluoresence, IF) and telomeres (fluorescence *in situ* hybridization, FISH), we found that cGAS are localized to telomeres and many other loci on chromosomes (Figs. 1C and S1D). Quantitative analysis revealed that ~84% telomeres are occupied by cGAS (Figs. 1D and S1E). The telomere-cGAS association was further demonstrated by ChIP assay coupled with slot blot using telomeric probe. These results showed that telomeric DNA are co-precipitated with endogenous cGAS in mitotic cells, but not in asynchronized cells (Figs. 1E, 1F and S1F–I). ChIP assay also identified DNA adjacent to telomeres termed “subtelomeres” that are co-precipitated with cGAS and detected by q-PCR using chromosome-specific primers (Fig. 1G and 1H). Altogether, these data revealed that cGAS occupies shelterin-deficient telomeres and subtelomeres during mitosis.

### Chromosome end-to-end fusion occurs in cGAS-deficient cells

**Figure 2:**
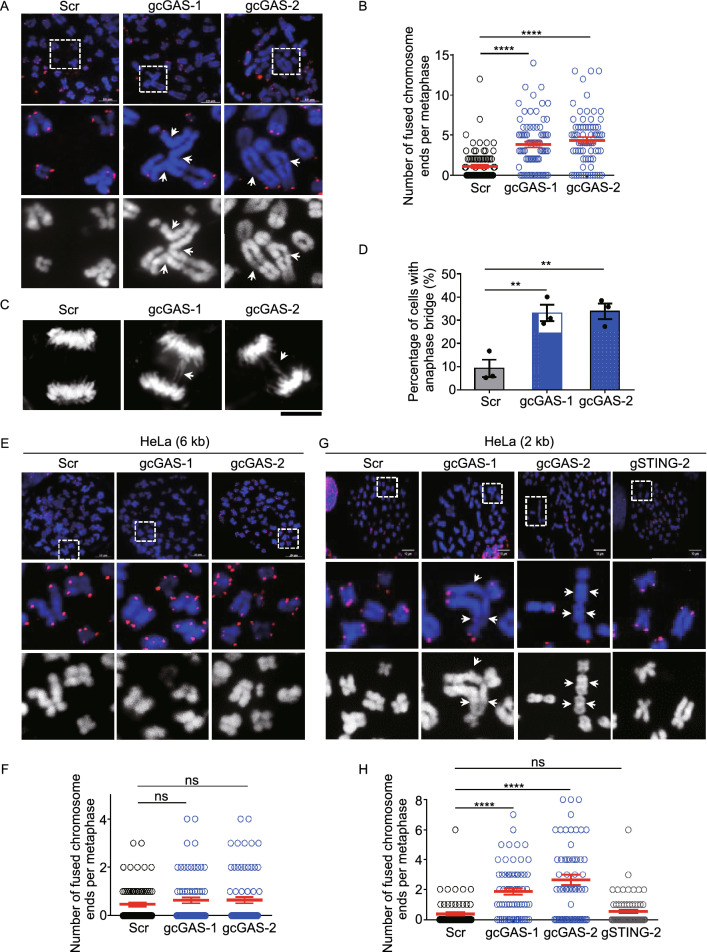
**cGAS protects short telomeres from being fused**. (A) FISH of telomeres on metaphase spreads to detect chromosome end-to-end fusions in U2OS. Cells were synchronized at G_1_/S, released for 8 h and then treated with colcemid for 12 h. cGAS was depleted by CRISPR/Cas9-based approach (gcGAS-1 or gcGAS-2, see method for details) and scramble sg-RNA (Scr) was used as a control. Fusion events were indicated with arrows. Scale bars, 10 μm. (B) Quantification of (A). The number of fused chromosome ends per metaphase was given (*n* ≥ 70 metaphase). All values are the average ± SEM of three independent experiments. (C) Detection anaphase bridge in control (Scr) and cGAS-depleted (gcGAS-1 or gcGAS-2) U2OS cells. Anaphase bridges were indicated with arrows. Scale bars, 10 μm. (D) Quantification of (C). The percentage of cells with anaphase bridge was calculated (*n* ≥ 76 cells). Only cells in anaphase were counted. All values are the average ± SEM of three independent experiments. (E) FISH of telomeres on metaphase spreads to detect chromosome end-to-end fusions in HeLa with average telomere length of 6 kb. The experiments were same as in (A). Scale bars, 10 μm. (F) Quantification of (E). The number of fused chromosome ends per metaphase was given (*n* ≥ 70 metaphase). All values are the average ± SEM of three independent experiments. (G) FISH of telomeres on metaphase spreads to detect chromosome end-to-end fusions in HeLa with average telomere length of 2 kb. The experiments were same as in (A). Cells with depletion of STING by CRISPR/Cas9-based approach (gSTING-2) was used as an additional control. Scale bars, 10 μm. (H) Quantification of (G). The number of fused chromosome ends per metaphase was given (*n* = 67 metaphase). All values are the average ± SEM of three independent experiments. Student’s unpaired two-tailed *t*-test was used to determine the statistical significance (**P < 0.01; ****P < 0.0001). See also Fig. S2

We then investigated the function of cGAS on telomeres/subtelomeres during mitosis. When cGAS was depleted by CRISPR/sgRNA in U2OS cells (Fig. S2A), we observed significantly increased frequency of chromosome end-to-end fusions on metaphase spreads (Figs. 2A, 2B, S2B and S2C). Consistently, cGAS-depletion led to significant increase of cells population (percentage) displaying anaphase bridge, a hallmark of chromosome fusion (Fig. 2C and 2D) (Fenech et al., 2011). Remarkably, we observed that fusion events predominantly occur between critically short telomeres showing limited or no telomeric signals on chromosome ends (Figs. 2A and S2D). Both average length of telomeres and number of critically short telomeres (including free ends) were not changed by depletion of cGAS (Fig. S2E), thus excluding the possibility that increased chromosome end-to-end fusions are caused by more telomere-free ends of chromosomes. Exactly the same phenomena were observed in cGAS-deficient VA13 cells, demonstrating the role of cGAS in preventing end-to-end fusion between critically short telomeres (Fig. S2F–J).

It is of note that both U2OS and VA13 are a telomerase-negative but ALT (Alternative Lengthening of Telomeres) positive cell line that is featured by a high heterogeneity of telomere length with extremely long and short telomeres (Cesare and Reddel, 2010). We also used telomerase positive HeLa cells that bear homogenous telomere lengths of ~6 kb with hardly any critically short telomeres (Fig. S2K), no increase of chromosome end-to-end fusions was detected on metaphase spreads upon depletion of cGAS (Figs. 2E, 2F and S2L). We thus hypothesized that cGAS deficiency-induced chromosome end-to-end fusion occurs exclusively on critically short telomeres. Indeed, when cGAS was depleted in subcloned HeLa cells with average telomere length of 2 kb (Fig. S2K and S2M) (Chen et al., 2015), significant increase of chromosome end-to-end fusions was detected (Fig. 2G and 2H). In contrast, depletion of STING in 2 kb-HeLa cells led to no increase of end-to-end fusions (Figs. 2G, 2H and S2N). These results in together with the fact that U2OS cells do not express STING strongly suggested that cGAS prevents the fusion of critically short telomeres in a STING-independent manner (Chen et al., 2017).

### c-NHEJ mediated telomere fusion is suppressed by cGAS during mitosis

**Figure 3:**
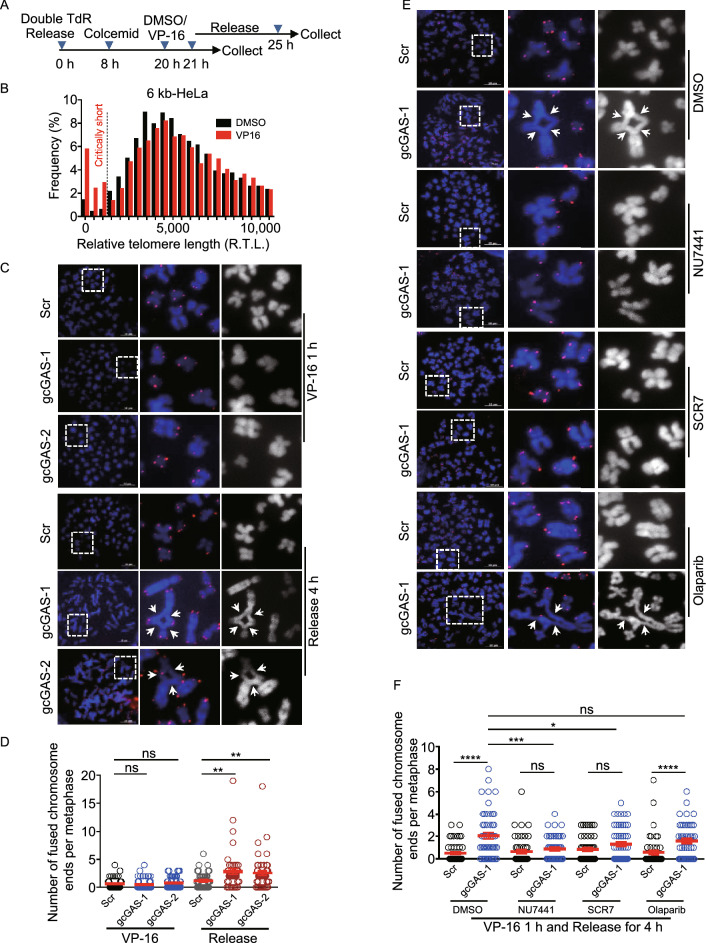
**cGAS inhibits c-NHEJ-mediated chromosome end-to-end fusion during mitosis**. (A) Time course of cell synchronization and treatment with VP-16. (B) The relative telomere length of control (DMSO) and VP-16 treated 6 kb-HeLa cells (VP-16) determined by q-FISH (*n* ≥ 3,000 chromosomes). (C) FISH of telomeres on metaphase spreads to detect chromosome end-to-end fusions in 6 kb-HeLa treated with VP-16. Control (Scr) or cGAS-depleted cells (gcGAS-1, gcGAS-2) were either harvested immediately following treatment (VP-16 1 h) or released for 4 h (Release 4 h) during mitosis. Fusion events were indicated with arrows. Scale bars, 10 μm. (D) Quantification of (C). The number of fused chromosome ends per metaphase was given (*n* ≥ 43 metaphase). All values are the average ± SEM of three independent experiments. (E) FISH of telomeres on metaphase spreads to detect chromosome end-to-end fusions in VP-16 treated 6 kb-HeLa cells in the presence of c-NHEJ (NU7441, SCR7) or alt-NHEJ (Olaparib) inhibitors. Control (Scr) or cGAS-depleted cells (gcGAS-1) were treated with VP-16 for 1 h, released for 4 h during mitosis, and then harvested for assay. Fusion events were indicated with arrows. Scale bars, 10 μm. (F) Quantification of (E). The number of fused chromosome ends per metaphase was given (*n* ≥ 48 metaphase). All values are the average ± SEM of three independent experiments. Student’s unpaired two-tailed *t*-test was used to determine the statistical significance (*P < 0.05; **P < 0.01; ***P < 0.001; ****P < 0.0001). See also Fig. S3

We then explored the mechanism underlying the suppression of chromosome end-to-end fusion by cGAS. First, we asked whether cGAS-deficiency-induced chromosome end-to-end fusion occurs during mitosis. To this end, cGAS-depleted 6 kb-HeLa cells were synchronized in mitosis by sequential treatment with thymidine and colcemid. The mitotic cells were then treated with VP-16 (etoposide, a topoisomerase II Inhibitor) for 1 h (Fig. 3A). We demonstrated that the treatment produces cohort of critical short telomeres (Fig. 3B), which is consistent with previous reports (Terasawa et al., 2014; Zhang et al., 2019a). The mitotic cells were either immediately harvested or released for 4 h in the presence of colcemid and then subjected to assay of metaphase spread and FISH (Figs. 3A and S3A). In contrast to immediately harvested cells that show no increase of end-to-end fusion, cells experiencing 4 h release during mitosis displayed significant increase of fusions (Fig. 3C and 3D), which predominantly occur at critically short telomeres (Fig. S3B).

Second, to explore a potential effect of shelterin proteins on chromosome end-to-end fusion during mitosis, their abundance on telomeres were re-examined in cGAS-deficient cells. ChIP data showed that cGAS depletion does not change the abundance of telomere-bound TRF1, TRF2 and POT1 in mitotic U2OS cells (Fig. S3C and S3D). Therefore, it is unlikely that end-to-end fusion is caused by the alteration in telomere-bound shelterin proteins.

Third, we examined what mechanism is responsible for cGAS-deficiency induced end-to-end fusion. The fusion could be potentially mediated by c-NHEJ or alt-NHEJ (Lieber, 2010). To this end, cGAS-deficient 6 kb-HeLa cells were synchronized in mitosis, treated with VP-16 for 1 h and released for 4 h in the presence of colcemid, meanwhile adding NU7441 (c-NHEJ/DNAPKcs inhibitor) or SCR7 (c-NHEJ/LIG4 inhibitor) or Olaparib (alt-NHEJ/PARP1 inhibitor) or B02 (HR/RAD51 inhibitor) (Fig. 3A). The results showed that both NU7441 and SCR7 but not Olaparib and B02 significantly suppress end-to-end fusions (Figs. 3E, 3F, S3E and S3F). This result strongly suggested that chromosome end-to-end fusion is mediated by c-NHEJ, but not alt-NHEJ.

### Telomeric DNA damage response (DDR) is suppressed by cGAS during mitosis

**Figure 4:**
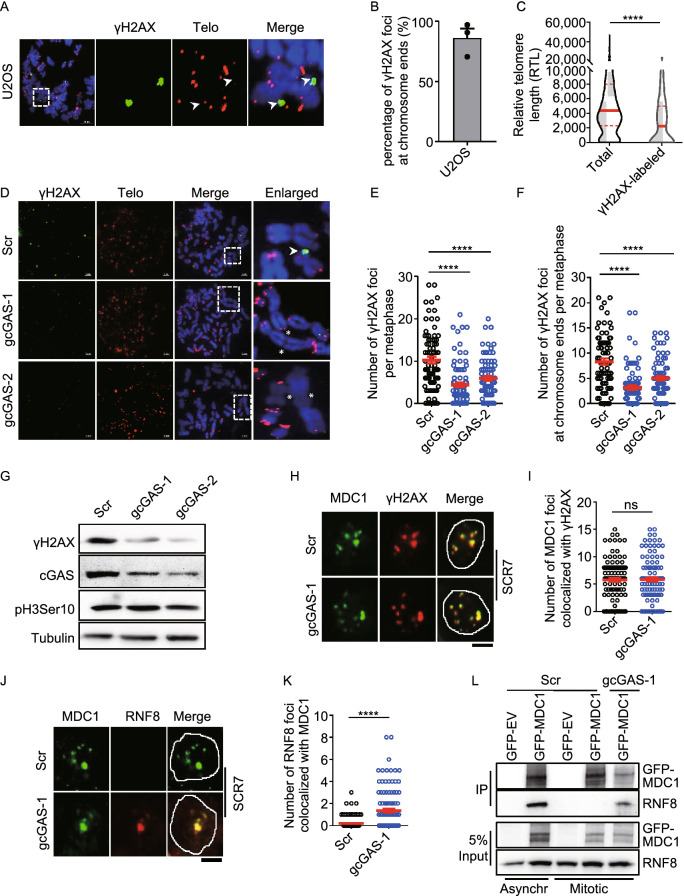
**The signaling cascade of DDR is activated at short telomeres but blocked by cGAS at the step of RNF8**. (A) IF and FISH performed on metaphase spreads of U2OS cells to visualize γH2AX foci and telomeres, respectively. Mitotic TIF were indicated with arrows. Scale bars, 10 μm. (B) Quantification of (A). The percentage of γH2AX foci colocalizing with telomeres/chromosome ends was given. All values are the average ± SEM of three independent experiments. (C) Quantification of (A). Relative lengths of telomeres colocalized with γH2AX were compared with that of total telomeres. (D) IF and FISH to visualize γH2AX foci and telomeres, respectively. Metaphase spreads were performed using control (Scr) or cGAS-deficient (gcGAS-1, gcGAS-2) U2OS cells. Mitotic-TIF and fusion events were indicated with arrows and stars, respectively. Scale bars, 5 μm. (E) Quantification of (D). The number of γH2AX foci per metaphase was given. All values are the average ± SEM of three independent experiments. (F) Quantification of (D). The number of γH2AX foci colocalized with telomeres/chromosome ends per metaphase was given. All values are the average ± SEM of three independent experiments. (G) Immunoblot analysis of γH2AX in control (Scr) and cGAS-deficient U2OS cells (gcGAS-1, gcGAS-2). Phosphorylated H3Ser10 was used as a marker for mitotic cells. (H) IF detection of γH2AX and MDC1 foci in control (Scr) or cGAS-deficient U2OS cells (gcGAS-1). Mitotic cells were cultured in the presence of SCR7. Scale bars, 5 μm. (I) Quantification of (H). The number of MDC1 colocalized with γH2AX foci per cell were calculated (*n* ≥ 100 cells). All values are the average ± SEM of three independent experiments. (J) IF detection of MDC1 and RNF8 foci in control (Scr) or cGAS-deficient U2OS cells (gcGAS-1). Mitotic cells were cultured in the presence of SCR7. Scale bars, 5 μm. (K) Quantification of (J). The number of MDC1 colocalized with RNF8 foci per cell were calculated (*n* ≥ 100 cells). All values are the average ±SEM of three independent experiments. (L) Co-IP assay to determine the interaction of GFP-MDC1 with RNF8 in control (Scr) and cGAS-deficient U2OS cells (gcGAS-1). Cells were cultured in the presence of SCR7. Asynchronous (Asynchr) or mitotic U2OS cells were immunoprecipitated with GFP-beads. GFP-EV was used as a control. Student’s unpaired two-tailed *t*-test was used to determine the statistical significance (****P < 0.0001). See also Fig. S4

It is believed that DNA damage repair including c-NHEJ is suppressed during mitosis (Giunta et al., 2010; Orthwein et al., 2014; Blackford and Stucki, 2020). Here, essential question is how telomeric c-NHEJ could occur during mitosis in cGAS-deficient cells. We observed that γH2AX foci, a marker of DDR, are frequently detected in mitotic U2OS cells (Fig. 4A). Meta-IF/FISH staining identified that more than 80% γH2AX foci are localized to chromosome ends (Fig. 4A and 4B). Strikingly, γH2AX foci were predominantly visualized at chromosome ends with short telomeres or undetectable telomeres (Fig. 4A and 4C), while the same phenomena were observed in VA13 cells (Fig. S4A–C). But, much fewer γH2AX foci were detected at chromosome ends in 6 kb-HeLa cells that have homogenous telomere length with much fewer critically short telomeres compared to U2OS and VA13 (Fig. S4D and S4E). The protein level of γH2AX in 6 kb-HeLa cells was also lower than that in U2OS and VA13 cells (Fig. S4F), consistent with the observation by IF. These results supported that DDR is activated at short telomeres during mitosis.

Interestingly, both number of γH2AX foci at chromosome ends and protein level of γH2AX were significantly reduced upon depletion of cGAS during mitosis (Fig. 4D–G). This may due to the fact that critically short telomeres undergo c-NHEJ mediated fusion in cGAS-deficient cells. Indeed, when c-NHEJ was inhibited by SCR7 in cGAS-deficient cells (Fig. S4G), the number of γH2AX foci at chromosome ends and protein level of γH2AX were back to the level of control cells (Fig. S4H–J). Therefore, to facilitate the study of mitotic DDR at chromosome ends in cGAS-deficient cells, following experiments were carried out in the presence of SCR7.

Because MDC1 is an immediate downstream factor of γH2AX (Stewart et al., 2003; Stucki et al., 2005), we first examined the recruitment of MDC1 onto γH2AX. It appeared that MDC1 are well colocalized with γH2AX regardless of the presence of cGAS (Fig. 4H and 4I), indicating that its recruitment is independent upon cGAS. RNF8 is a key player in DDR that is recruited by interacting with MDC1 (Huen et al., 2007; Kolas et al., 2007; Mailand et al., 2007). No RNF8 foci were detected in mitotic U2OS cells, however, depletion of cGAS leads to formation of RNF8 foci, which are colocalized with MDC1 (Fig. 4J and 4K). MDC1-RNF8 interaction was further confirmed by co-IP experiment, in which RNF8 was precipitated by GFP-MDC1 during mitosis only if cGAS was depleted (Fig. 4L). Taken together, these results strongly suggested that cGAS inhibits the recruitment of RNF8 to DNA damage sites (γH2AX). As a result of failure in recruiting RNF8, there was no 53BP1 foci (a downstream factor of RNF8) detected in mitotic U2OS cells, but depletion of cGAS led to formation of 53BP1 foci that were colocalized with γH2AX foci (Fig. S4K and S4L).

### Recruitment of CDK1 by cGAS to chromosome ends

**Figure 5:**
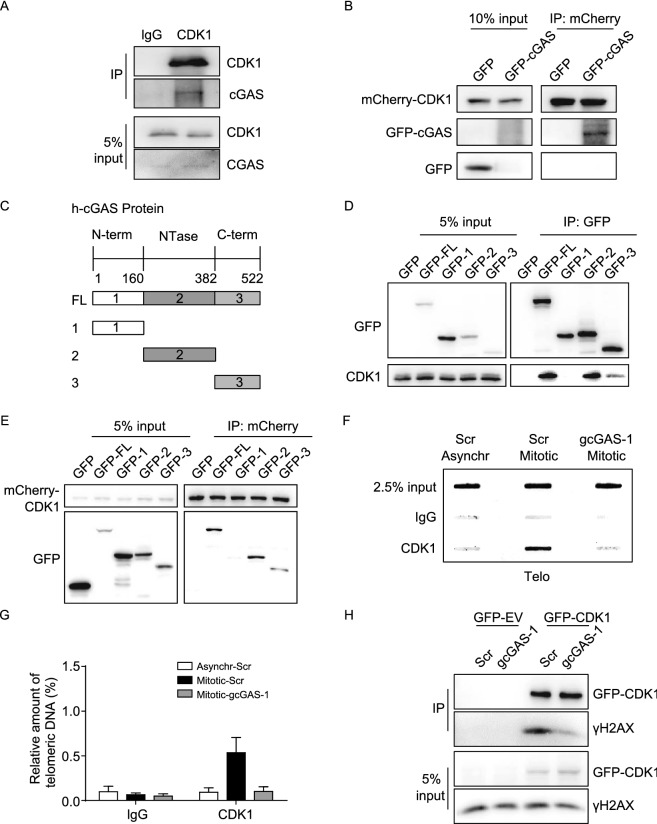
**cGAS interacts with CDK1**. (A) Co-IP assay to determine the interaction of endogenous CDK1 with cGAS in mitotic U2OS cells. IgG was used as a negative control. Cells were synchronized in mitosis as described above. (B) *In vitro* pull-down assay using exogenously purified GFP-cGAS protein and mCherry-CDK1 protein to determine the direct interaction of CDK1 with cGAS *in vitro*. GFP was used as a negative control. (C) Schematic diagram showing the domains of human cGAS protein. (D) Co-IP assay to determine the domain of cGAS that interacts with CDK1. 293T cells transfected with GFP-tagged full-length (GFP-FL) or its domain (GFP-1, -2 or -3) were treated with colcemid for 16 h and subjected to immunoprecipitation with GFP-beads. (E) Reverse-co-IP assay to determine the domain of cGAS that interacts with CDK1. 293T cells transfected with mCherry-CDK1 and GFP-tagged full-length (GFP-FL) or its domain (GFP-1, -2 or -3) were treated with colcemid for 16 h and subjected to immunoprecipitation with mCherry-beads. (F) ChIP assay coupled with slot blot and hybridization with telomeric probe to determine the interaction between CDK1 and telomeres in control (Scr) or cGAS-deficient (gcGAS-1) U2OS cells. Cells were either asynchronized or synchronized in mitosis as described above. (G) Quantification of (F). The relative amount of enriched telomeric DNA was calculated (ChIP/Input, %). All values are the average ± SEM of three independent experiments. (H) Co-IP assay to determine the interaction of GFP-CDK1 with γH2AX in control (Scr) or cGAS-deficient (gcGAS-1) U2OS cells. Cells were synchronized in mitosis as described above. See also Fig. S5

It has been reported previously that both RNF8 and 53BP1 are deactivated by CDK1-mediated phosphorylation during mitosis (Orthwein et al., 2014). In context of telomeres, cGAS may participate in prepositioning CDK1 to chromosome ends where it executes the function. The physical interaction between cGAS and CDK1 was examined by co-IP. The results showed that both endogenous and exogenously expressed CDK1 are able to precipitate cGAS in mitotic U2OS cells (Figs. 5A and S5A). Interaction between CDK1 and cGAS was also observed in HeLa cells (Fig. S5B). Furthermore, CDK1-cGAS interaction is not mediated by DNA because pretreatment with DNase I does not affect the amount of CDK1 precipitated by GFP-cGAS (Fig. S5C and S5D). Importantly, we examined the direct interaction between CDK1 and cGAS by an *in vitro* pull-down assay using purified GFP-cGAS and mCherry-CDK1 (Fig. 5B). The result showed that mCherry-CDK1 could directly bind with GFP-cGAS *in vitro*. Our results are consistent with previous excellent finding that CDK1 interacts with and phosphorylates cGAS during mitosis (Zhong et al., 2020).

cGAS consists of N-terminal, NTase and C-terminal domains (Fig. 5C) (Sun et al., 2013). To investigate which domain is responsible for interacting with CDK1, GFP-fused domains were individually expressed in 293T cells and their interaction with CDK1 was examined by co-IP using GFP antibody-conjugated beads. We found that CDK1 showed strong interaction with NTase domain, and weak interaction with C-terminal domain of cGAS, while it did not interact with N-terminal domain of cGAS (Fig. 5D). The result was confirmed by reverse co-IP, in which GFP-NTase is a major product precipited by mCherry-tagged CDK1 (Fig. 5E).

It is speculated that cGAS-CDK1 interaction would bring CDK1 to telomeres and DNA damage sites. To test it, CDK1-ChIP was performed to examine the localization of CDK1 on telomeres in the presence or absence of cGAS. Our results showed that CDK1 is able to precipitate telomeric DNA, whereas depletion of cGAS abolishes the precipitation (Fig. 5F and 5G). Similarly, co-IP assay demonstrated that γH2AX are co-precipitated with GFP-tagged CDK1 in mitotic U2OS cells, but depletion of cGAS leads to significant decrease of γH2AX precipitated (Fig. 5H), suggesting that CDK1 are positioned to DNA damage sites (γH2AX) in a cGAS-dependent manner.

### cGAS is required for replicative senescence

**Figure 6:**
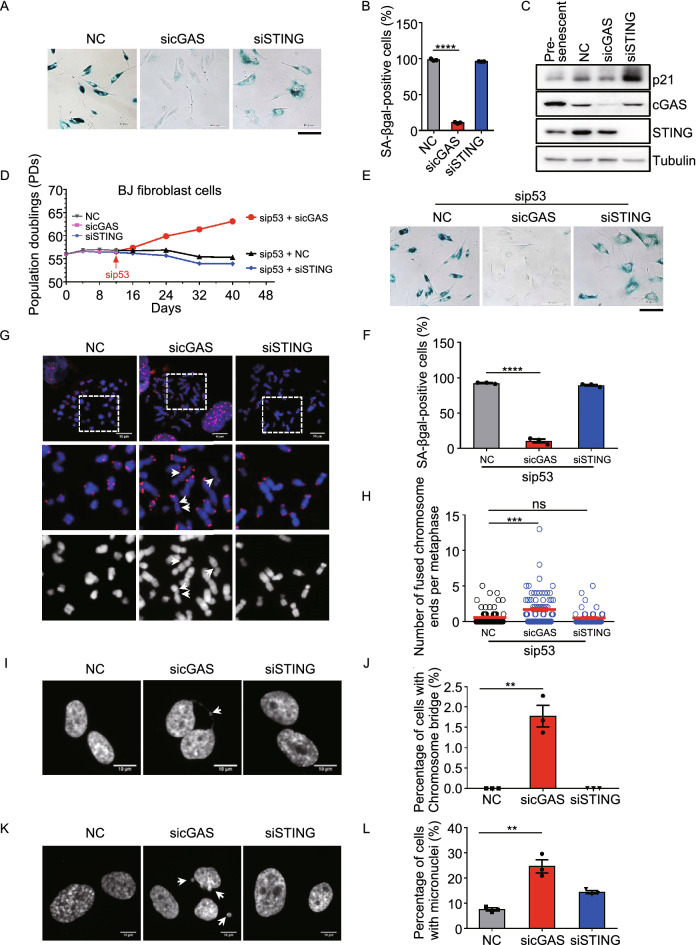
**cGAS promotes replicative senescence to prevent genome instability**. (A) SA-β-gal staining of pre-senescent BJ fibroblast cells transfected with si-scramble (NC) or sicGAS or siSTING. After transfection, cells were cultured for 16 days and subjected to SA-β-gal staining. Scale bars, 100 μm. (B) Quantification of (A). The percentage of SA-β-gal positive cells was calculated (*n* ≥ 100 cells). All values are the average ± SEM of three independent experiments. (C) Immunoblot analysis of p21 in pre-senescent BJ fibroblast cells transfected with si-scramble (NC), sicGAS or siSTING. After transfection, cells were cultured for 12 days and subjected to immunoblot. Pre-senescent BJ cells was used as a control. (D) Growth curve of pre-senescent BJ fibroblast cells sequentially transfected with si-scramble (NC) or sicGAS or siSTING for 12 days and then with sip53. Corresponding siRNA was transfected every 4 days. (E) SA-β-gal staining of pre-senescent BJ fibroblast cells sequentially transfected with si-scramble (NC) or sicGAS or siSTING for 12 days and then with sip53 for 4 days. Scale bars, 100 μm. (F) Quantification of (E). The percentage of SA-β-gal positive cells was calculated (*n* ≥ 100 cells). All values are the average ± SEM of three independent experiments. (G) FISH of telomeres on metaphase spreads to detect chromosome end-to-end fusions in cGAS (or STING) and p53-double knocked down BJ fibroblast cells. Pre-senescent cells were treated with indicated siRNA and sip53 for 12 days and subjected to FISH. Fusion events were indicated with arrows. Scale bars, 10 μm. (H) Quantification of (G). The number of fused chromosome ends per metaphase was calculated (*n* ≥ 50 metaphase). All values are the average ± SEM of three independent experiments. (I) Detection of chromosome bridges in control (NC), cGAS (sicGAS) or STING (siSTING) deficient BJ cells. Chromosome bridge was indicated with arrow. Scale bars, 10 μm. (J) Quantification of (I). The percentage of cells with chromosome bridge was calculated (*n* ≥ 100 cells). All values are the average ± SEM of three independent experiments. (K) Detection of micronucleis (MNs) in control (NC), cGAS (sicGAS) or STING (siSTING) deficient BJ cells. MNs were indicated with arrows. Scale bars, 10 μm. (L) Quantification of (K). The percentage of cells with micronuclei (*n* ≥ 100 cells) was calculated. All values are the average ± SEM of three independent experiments. Student’s unpaired two-tailed *t*-test was used to determine the statistical significance (**P < 0.01; ***P < 0.001; ****P < 0.0001). See also Fig. S6

For human normal cells, critically short telomeres trigger replicative senescence by activating telomeric DDR that stimulates p53/p21-mediated cellular senescence (Harley et al., 1990; Shay et al., 1991; Hemann et al., 2001). However, the fusion between critically short telomeres may quench DDR (Fig. 4D–G), suggesting the possibility that the replicative senescence would be prevented by short telomere fusion caused by depletion of cGAS. We tested this possibility using pre-senescent BJ cells. Firstly, we found that cGAS localized to telomeres and many other loci on chromosomes in pre-senescent BJ cells (Fig. S6A and S6B). Secondly, cGAS or STING was depleted in human pre-senescent BJ primary fibroblast cells. After 16 days of culturing, cGAS-deficient cells expressed low level of p21 and were negative for β-gal staining as compare with control, indicating the failure in undergoing senescence (Fig. 6A–C). In contrast, STING-deficient cells underwent cellular senescence, displaying increased level of p21 and positivity for β-gal staining (Fig. 6A–C). p53 is the key regulator that initiates replicative senescence (Shay et al., 1991). p53 mutation or deficiency allows cells to overcome replicative senescence and continue to proliferate (Wei and Sedivy, 1999). Unlike senescent control and STING-deficient BJ cells, cGAS-deficient BJ cells re-started proliferation upon further depletion of p53 at day 12 (Figs. 6D and S6C). Further experiment showed that cGAS/p53 double-depleted cells did not undergo senescence, which is different from NC/p53 or STING/p53 double-depleted cells (Fig. 6E and 6F). Taken together, these results proved that cGAS-deficient cells did not undergo senescence and depletion of p53 enabled cGAS-deficient cells to re-start the proliferation.

In order to acquire the mitotic cells, we simultaneously knocked down p53 coupled with cGAS or STING in pre-senescent BJ fibroblast cells. We carried out metaphase spread to examine whether chromosome end-to-end fusion occurs in p53/cGAS double-depleted BJ cells. Indeed, p53/cGAS, but not p53/STING double-depleted cells showed increase of chromosome end-to-end fusions (Fig. 6G and 6H). Moreover, the fusion predominantly occurs between critically short telomeres (Figs. 6G and S6D). To exclude the potential role of HR in cGAS prevention of replicative senescence, we knocked down the essential gene of HR (RAD51) and NHEJ (LIG4) respectively in combination with transfection of sicGAS or siSTING. The results showed that the decrease of senescent BJ cells after cGAS depletion was counteracted by knockdown of LIG4 but not RAD51 (Fig. S6E–G). These results identified cGAS promotes replicative senescence by inhibiting NHEJ but not HR. In addition, we observed that cGAS-deficient, but not senescent control or STING-deficient BJ cells display high frequency of chromosome bridge and micronuclei (Fig. 6I–L), indicating the genome instability induced by chromosome end-to-end fusions (Fenech et al., 2011).

## DISCUSSION

Critically short telomeres are recognized as double-stranded breaks (DSBs) that stimulate persistent DDR at chromosome ends. However, these DSBs and DDR do not lead to DNA-repair and chromosome end-to-end fusion in human normally senescent cells or some of the specific cancer cells such as ALT cancers. Here, we found that during mitosis, cGAS occupies multiple loci on chromosomes including telomeric/subtelomeric DNA, wherein it blocks DDR and NHEJ pathway by interacting with and recruiting CDK1. This function of cGAS, which is independent of the cGAS-STING axis, provides new insight into how human cells escape undesired DNA-repair when telomeres become critically short in order to trigger replicative senescence.

### DDR on short telomeres during mitosis

ALT cancers such as U2OS and VA13 cells that bear a high heterogeneity of telomere length are a good model to study DDR induced by short telomeres. In addition, U2OS cells lack the expression of STING (Chen et al., 2017), providing a clean system to study the function of cGAS on DDR. Because a vast majority of TRF1, TRF2 and POT1 departs from telomeres during mitosis (Fig. 1A and 1B), it is speculated that shelterin complex on its own would be insufficient to protect telomeres. Our previous study showed that t-loops re-fold immediately following telomere replication, indicating that telomeres are in the form of t-loops during mitosis that could prevent the activation of DDR at chromosome ends (Zhang et al., 2019b). However, critically short telomeres may not be able to form t-loops. In consistent with this, we observed that mitotic γH2AX are predominantly localized to chromosome ends with short telomeres in U2OS and VA13 cells (Figs. 4A–C and S4A–C). Similarly, activation of DDR at critically short telomeres has been observed in human primary cells when reaching replicative senescence (Zou et al., 2004). However, activated DDR does not lead to subsequent DNA-repair and chromosome end-to-end fusion. Here, we hypothesized that the signal transduction of DDR at critically short telomeres is blocked by the cGAS-CDK1 axis.

### cGAS and chromosome end protection

Intriguingly, cGAS associating with telomeres coincides with the departure of shelterin proteins from telomeres, implying that cGAS may serve as a substitute of shelterin/TRF2 for protecting chromosome ends during mitosis. Indeed, depletion of cGAS leads to chromosome end-to-end fusion in both human cancer and normal cells (Figs. 2A–D and 6G–J). However, cGAS and TRF2-mediated end protection are different in many aspects. Firstly, low sequence specificity allows cGAS to bind not only telomeres, but also subtelomeres, rendering them a capacity to protect critically short telomeres. Second, the cGAS-CDK1 axis disrupts a signal transduction of DDR by deactivation of RNF8, whereas TRF2 inhibits the activation of DDR by mainly suppressing ATM (Denchi and de Lange, 2007). Third, the cGAS-CDK1 axis prevents the fusion of critically short telomeres during mitosis. In contrast, TRF2 protects all telomeres, conceivably during G_1_ and S phase of cell cycle.

### cGAS is involved in DNA damage repair

The function of cGAS on regulating DNA damage repair has been recently identified. For instance, Liu et al. reported that cGAS is phosphorylated and transported from cytosol into nucleus where it impedes homologous recombination (HR) via specific interaction with DNA repair proteins PARP1 (Liu et al., 2018). Moreover, it has been revealed that cGAS is a chromatin-bound protein that inhibits HR-mediated DNA repair by compaction of bound DNA (Jiang et al., 2019). Hence, the role of cGAS in HR is well studied in detail. Here we further found cGAS also plays a key role in NHEJ of short telomere during mitosis. In our work, we found synchronization of cGAS-depleted cells with critical short telomere in mitosis leads to NHEJ. In this circumstance, both short telomere and mitosis synchronization are essential for NHEJ occurrence. Consistently, we observed that depletion of cGAS induced NHEJ during mitosis in 2 kb-HeLa but not 6 kb-HeLa cells (Fig. 2E–H).

Here, we proposed that during mitosis chromosome-associated cGAS recruits CDK1 to critically short telomeres, wherein CDK1 blocks signal transduction of DDR and NHEJ by deactivation of RNF8. Previously, excellent work done by Orthwein et al. revealed that CDK1 inhibits mitotic DNA damage repair by phosphorylating RNF8 and 53BP1 (Orthwein et al., 2014). However, it is unclear how CDK1 is recruited onto DNA damage sites to execute its function. It is unlikely that CDK1 phosphorylates all RNF8 and 53BP1 during mitosis. Our results finished the last piece of puzzle by showing that depletion of cGAS impairs CDK1’s enrichment at DNA damage sites/telomeres leading to resumption of DDR, i.e., recruitment of RNF8/53BP1, and chromosome end-to-end fusion (Figs. 5F–H and 4J–L). This finding emphasized the role of positioning CDK1 to location on demand. Although cGAS-dependent enrichment of CDK1 was studied in the scenario of telomeres, this mechanism should not be restricted to telomeres, because cGAS occupies multiple loci on chromosomes (Figs. 1C and S1D) (Gentili et al., 2019), wherein DNA damages may occur. Further investigation is needed to explore this possibility.

### cGAS is required for successful replicative senescence

It is demonstrated that an average of 5 dysfunctional telomeres is need for permanent cell cycle arrest and senescence (Kaul et al., 2011). It is also believed that critically short telomeres induce DNA damage signal that stimulates the expression of p21, which initiates cellular senescence when reaching a critical level (Nassrally et al., 2019). In this theory, fusion between critically short telomeres would quench DDR and reduce the level of p21. Indeed, we observed that depletion of cGAS in pre-senescent BJ fibroblast cells leads to lower expression of p21 that is associated with fewer cells undergoing senescence (Fig. 6A–C). The dramatic increase of p21 after knockdown of STING suggests an important role of STING in replicative senescence, which worth further exploration. Interestingly, though cGAS-depleted cells are negative for β-gal, only ~7% of population is positive for EdU-staining, indicating DNA synthesis but the arrested cell proliferation (Figs. S6H, S6I and 6D). Upon depletion of p53, cells immediately re-started the proliferation (Fig. 6D). It should be noted that the cGAS-STING axis is previously reported to promote stress-induced senescence by stimulating SASP (senescence-associated secretory phenotype) (Dou et al., 2017; Glück et al., 2017; Yang et al., 2017). This mechanism is distinct from cGAS’s function reported, which is independent upon STING. Nonetheless, both highlight the important roles of cGAS during cellular senescence.

Collectively, cGAS plays a key role in immune system via the cGAS-STING axis and also affects DNA damage repair. Dysregulations in the immune system and DNA damage repair led to many human disorders including inflammatory diseases, aging-associated diseases and cancers. This work brings up new perspective for future research in regard to how cGAS is regulated during cell cycle process and its role in disease.

## MATERIALS AND METHODS

### Cell culture, transfection

U2OS, VA13, HeLa and 293T cells were obtained from American Type Culture Collection (Manassas, VA). BJ fibroblast cells were obtained from the Cell Resource Center of Peking Union Medical College. All cells were grown in DMEM (GIBCO) supplemented with 10% fetal bovine serum (GIBCO) and 100 U per Ml penicillin/streptomycin (GIBCO). Cells were cultured at 37  °C with 5% CO_2_. Plasmid DNA was transiently transfected into 293T or HeLa cells using the PEI method: plasmid DNA was mixed with PEI and incubated for 15 min. The mixture was added to culture medium where cells reach 30%–40% of confluence. After incubation of 6 h, the medium was replaced with fresh medium and cells were incubated for additional 72 h.

### Plasmids

cDNA of CDK1 were a kind gift from Dr. Songyang’ s lab at Sun Yat-sen university, which was cloned into pC1-GFP/mCherry or pLVX-Puro/Hygro plasmids. cGAS was cloned into pC1-GFP or pLVX-Puro/Hygro plasmid. PLenti CMV/TO GFP-MDC1 was purchased from Addgene (Plasmid #26285).

### Cell treatments

For the purpose indicated in main text, cells were treated with VP-16 (2 μmol/L, MCE); NU7441 (0.5 μmol/L, Selleck); SCR7 (20 μmol/L, Selleck); Olaparib (10 μmol/L, Selleck); B02 (27.4 μmol/L, Selleck); Colcemid (1 μg/mL, MCE).

### Gene silencing and knockout

siRNAs were transfected into target cells using Lipofectamine® RNAiMAX Transfection Reagent (Invitrogen), according to the manufacturer’s instructions. siRNA (cGAS: 5′-GCAUGCAAAGGAAGGAAATdTdT-3′; STING: 5′- CCCGGAUUCGAACUUACAAdTdT-3′; p53: 5′-GCAUGAACCGGAGGCCCAUdTdT-3′; RAD51: 5′-GGAAGAAGCUGGAUUCCAUdTdT-3′; LIG4: 5′-GCUUGGUGUUAGUCAGCAAdTdT-3′), were provided by GenePharma Co., Ltd (Suzhou, China). The scrambled sequence was used as a control. For depletion of cGAS and STING genes, the sgRNA sequence (gcGAS-1: 5′-CACCGGCACGTCCCAGGGCCCGGG-3′; gcGAS-2: 5′- CACCGATGATATCTCCACGGCGGC-3′; gSTING-2: 5′-CACCTACTCCCTCCCAAATGCGGT-3′) were cloned into lenti-CRISPRv2 consisting of Flag-Cas9. Lentivirus was packaged in 293T cells using PEI transfection. Viral supernatants were collected to infect target cells. After selection with puromycin (1 μg/mL) for 72 h, cells were ready for usage. Lenti-CRISPRv2 with scrambled sequence was used as a control.

### Cell cycle synchronization

VA13, U2OS and HeLa cells were synchronized at G_1_/S using the “double thymidine”, as previously described with a minor modification (Zhao et al., 2009). Briefly, cells were incubated with thymidine (2 mmol/L) for 19 h, washed three times with PBS, and released into fresh medium for 10 h. Thymidine (2 mmol/L) was then added for 14 h, washed with PBS for three times, then released into fresh medium for 8 h. Colcemid (1 μg/mL) was added and incubated for 12 h to synchronize the cells in mitosis.

### Immunoblotting

Immunoblotting assays were carried out according to standard protocols. Antibodies used are as follows: MDC1 (1:5,000, ab11169, Abcam), RNF8 (1:1000, sc-271462, Santa Cruz), cGAS (1:1000, 15102, CST), STING (1:1000, 13647, CST), CDK1 (1:2,000, ab18, Abcam), GFP (1:2,000, D110008, Sangon), pH3Ser10 (1:1000, 9701, CST), γH2AX (1:2,000, 9718, CST), p53 (1:1000, sc-126, Santa Cruz), p21 (1:1000, sc-6246, Santa Cruz), mCherry (1:2,000, 26765-1-AP, Proteintech), Tubulin (1:5,000, 66031-1-Ig, Proteintech), GAPDH (1:5,000, 60004-1-Ig, Proteintech) and HRP-conjugated anti-rabbit or anti-mouse (KPL, Inc).

### Immunofluorescence (IF) and immunofluorescence *in situ* hybridization (IF-FISH)

For IF experiments, cells plated on coverslips were fixed with 4% paraformaldehyde for 15 min, permeabilized with 0.2% Triton X-100 for 10 min and blocked with 5% goat serum for 1 h. The coverslips were incubated sequentially with primary antibody at 4 °C overnight and fluorescence-labeled secondary antibody for 1 h at room temperature. For IF-FISH, the coverslips were then hybridized with PNA probe (TelG-Cy3, Panagene). Coverslips were stained with DAPI (Vector Laboratories) and visualized using fluorescence microscopy.

Antibodies used are as follows: anti-cGAS (1:100, CST), anti-53BP1 (1:2,000, NB100-304, Novus), MDC1 (1:400, ab11171, Abcam), anti-RNF8 (1:100, Santa Cruz), anti-CDK1 (1:200, Abcam), anti-γH2AX antibody (1:400, CST), anti-γH2AX antibody (1:400, ab26350, Abcam).

### Telomere FISH and Metaphase IF-FISH (Meta-IF/FISH)

For telomere FISH experiments, cells were synchronized at metaphase. Cells were swelled in 0.2% KCl and 0.2% trisodium citrate hypotonic buffer at room temperature for 7 min, then cytocentrifuged onto coverslips at 2,000 rpm for 2 min. Cells were fixed with 4% paraformaldehyde for 15 min and permeabilized with KCM buffer (120 mmol/L KCl, 20 mmol/L NaCl, 10 mmol/L Tris-HCl (pH 7.5) and 0.1% Triton X-100) at room temperature for 10 min. Cells were dehydrated with 75, 95, 100% ethanol and incubated with PNA probe at 85 °C for 5 min, followed by hybridization at 37 °C overnight. The coverslips were washed, stained with DAPI (Vector Laboratories) and visualized using fluorescence microscopy.

For Meta-IF/FISH assay, after incubation with PNA probe, coverslips were fixed with 4% paraformaldehyde for 10 min, washed with PBS, blocked with 100 μg/mL DNase-free RNase A (Sigma) in blocking buffer (5% Goat Serum in PBS) for 1 h at 37 °C. Slides were incubated with primary antibody at 4 °C overnight, washed with PBST, and incubated with secondary antibody for 1 h at room temperature. Antibodies used were as follows: anti-cGAS (1:100, CST), anti-γH2AX antibody (1:400, CST).

### Chromatin immunoprecipitation (ChIP), DNA Slot blot and ChIP q-PCR

ChIP assays were performed as previously described (Wu et al., 2014). Antibodies used were as follows: anti-cGAS (CST), anti-CDK1 (Abcam), anti-TRF1 (GTX77605, GeneTex), anti-TRF2 (05-521, Millipore), anti-POT1 (NB500-176, Novus), anti-IgG (Millipore).

ChIP DNA were subjected to slot blot and hybridization with biotin-labeled telomeric G-rich probe or Alu probe. Probe sequences are: Telo probe 5′-TTAGGGTTAGGGTTAGGGT-3′; Alu probe 5′-GGCCGGGCGCGGTGGCTCACG CCTGTAATCCCAGCA-3′. ChIP DNA were also used for q-PCR to determine subtelomeric DNA. Primers used were listed in Table S1.

### Co-immunoprecipitation

Cell lysates were prepared using RIPA buffer (1% NP-40, 0.25% sodium deoxycholate, 50 mmol/L Tris-HCl (pH 7.4), 150 mmol/L NaCl, 1 mmol/L EDTA, 1 mmol/L Na_3_VO_4_, 10 mmol/L Na_4_P_2_O_7_, 1 mmol/L NaF) containing phosphatase and proteinase inhibitors (Abmole). Endogenous immunoprecipitation was performed by incubating U2OS or HeLa cell lysates with primary antibody overnight, incubating with Protein A/G beads (Santa Cruz) for 4 h, washing with RIPA buffer for three times and elution. Following primary antibodies were used: CDK1 (Abcam), cGAS (CST). DNase I were purchased from Takara (Beijing, China). GFP-tagged or mCherry-tagged proteins were immunoprecipitated using GFP-beads or mCherry-beads (Kang Ti, China).

### Protein expression and purification

The expressing plasmids encoding cGAS conjugated EGFP tag were transformed into *E. coli* BL21. Freshly transformed cells were grown in LB containing Kanamycin (50 µg/mL) at 37 °C for about 12 h. After induction with 1 mmol/L IPTG at 37 °C for 6 h, cells were harvested by centrifugation and resuspended in His-lysis buffer (50 mmol/L Tris-HCl (pH 7.5), 500 mmol/L NaCl, 20 mmol/L imidazole, 0.035% β-ME, 5% glycerol and protease inhibitors). Cells were lysed by sonication on ice and centrifuged to remove debris. The supernatant was purified by incubation with Ni-NTA agarose beads overnight at 4 °C. Ni-NTA beads were then washed with wash buffer, and proteins were eluted with elution buffer (50 mmol/L Tris-HCl (pH 7.5), 500 mmol/L NaCl, 250 mmol/L imidazole, 0.035% β-ME, 5% Glycerol). Eluted protein was subjected to gel filtration on a 16/600 G200 Superdex column in 20 mmol/L Tris-HCl (pH 7.5), 300 mmol/L NaCl, 1 mmol/L DTT. The peak sample was concentrated, measured and stored at −80 °C before use.

### *In vitro* pull-down assay

GFP-cGAS protein was incubated with mCherry-CDK1 protein purified from 293T cells and mCherry-Beads in RIPA buffer (1% NP-40, 0.25% sodium deoxycholate, 50 mmol/L Tris-HCl (pH 7.4) and 150 mmol/L NaCl) overnight at 4 °C, then washed four times with RIPA buffer. The samples were analyzed by immunoblotting using following antibodies: anti-GFP (Sangon), anti-mCherry (Proteintech).

### SA-β-gal staining

The senescence-associated beta-galactosidase (SA-β-gal) staining assay was performed using an SA-β-gal staining kit (Beyotime, China) following the manufacturer’s instructions.

### Statistics

GraphPad Prism 8 was used for statistical analysis. Results are shown as mean ± SEM and the unpaired Student’s two-tailed *t*-test was used to determine the statistical significance (*P < 0.05; **P < 0.01; ***P < 0.001;****P < 0.0001).

## Supplementary Information

Below is the link to the electronic supplementary material.

Supplementary file1 (PDF 1542 kb)

Supplementary file2 (XLSX 11 kb)
