# Supplementary material for: cGAS guards against chromosome end-to-end fusions during mitosis and facilitates replicative senescence
Source: Protein Cell. 2021 Oct 22;13(1):47–64. doi: 10.1007/s13238-021-00879-y (PMC8776970; doi:10.1007/s13238-021-00879-y)
Supplement: Supplementary file 1 — Supplementary file1 (PDF 1542 kb) [file 13238_2021_879_MOESM1_ESM.pdf]

Figure S1

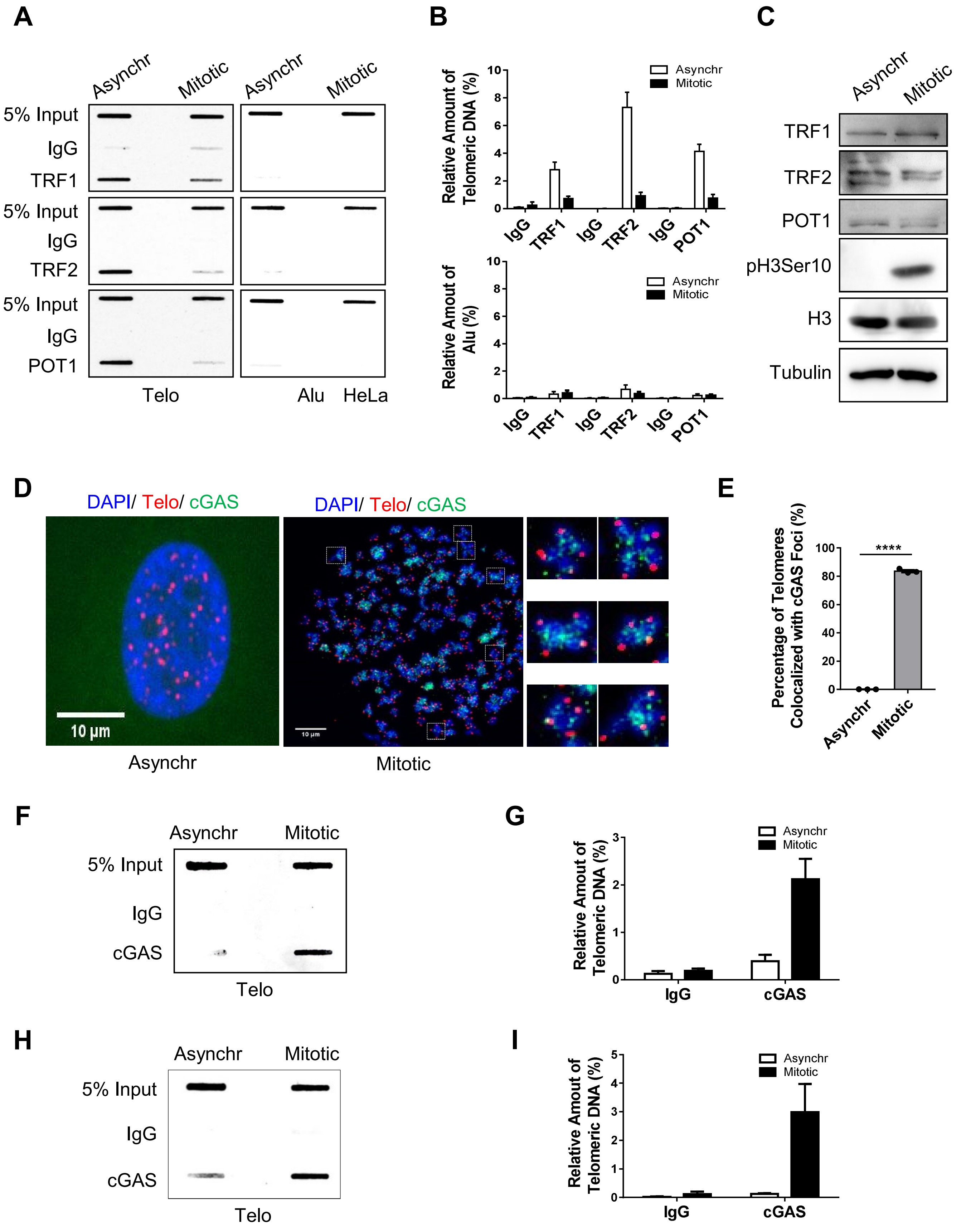

A
Asynchr
Mitotic
Asynchr
Mitotic
5% Input
IgG
TRF1
5% Input
IgG
TRF2
5% Input
IgG
POT1
Telo
Alu
HeLa
B
Relative Amount of Telomeric DNA (%)
Asynchr
Mitotic
IgG
TRF1
IgG
TRF2
IgG
POT1
Relative Amout of Alu (%)
C
Asynchr
Mitotic
TRF1
TRF2
POT1
pH3Ser10
H3
Tubulin
D
DAPI/ Telo/ cGAS
10 μm
Asynchr
Mitotic
E
Percentage of Telomeres Colocalized with cGAS Foci (%)
****
Asynchr
Mitotic
F
Asynchr
Mitotic
5% Input
IgG
cGAS
Telo
G
Relative Amout of Telomeric DNA (%)
Asynchr
Mitotic
IgG
cGAS
H
Asynchr
Mitotic
5% Input
IgG
cGAS
Telo
I
Relative Amout of Telomeric DNA (%)
Asynchr
Mitotic
IgG
cGAS

Figure S1.

(A) ChIP analysis of TRF1, TRF2 and POT1 associating with telomeres in asynchronous (Asynchr) or mitotic HeLa cells. Cells were either asynchronous or synchronized at G1/S, released for 8 h and then treated with colcemid for 12 h. ChIP enriched DNA was used for slot blot and hybridization using telomeric G-rich probe or Alu probe.

(B) Quantification of A. The relative amount of enriched telomeric DNA was calculated (ChIP / Input, %). Alu was used as a control. All values are the average $\pm$SEM of three independent experiments.

(C) Immunoblot analysis TRF1, TRF2 or POT1 in asynchronous (Asynchr) and mitotic U2OS cells. Phosphorylated H3Ser10 was used as a marker for mitotic cells.

(D) Visualization of endogenous cGAS and telomeres in asynchronous (Asynchr) or mitotic HeLa cells. Cells were treated as in A and then subjected to metaphase spread followed by IF/FISH. Scale bars, 10 μm.

(E) Quantification of D. Percentage of telomeres colocalizing with cGAS foci was calculated. All values are the average $\pm$SEM of three independent experiments.

(F) ChIP analysis of cGAS associating with telomeres in asynchronous or mitotic HeLa cells.

(G) Quantification of F. The relative amount of enriched telomeric DNA was calculated (ChIP / Input, %). All values are the average $\pm$SEM of three independent experiments.

(H) ChIP analysis of cGAS associating with telomeres in asynchronous or mitotic VA13 cells.

(I) Quantification of H. The relative amount of enriched telomeric DNA was calculated (ChIP / Input, %). All values are the average $\pm$SEM of three independent experiments. The unpaired Student's two-tailed t-test was used to determine the statistical significance (****P<0.0001).

Figure S2

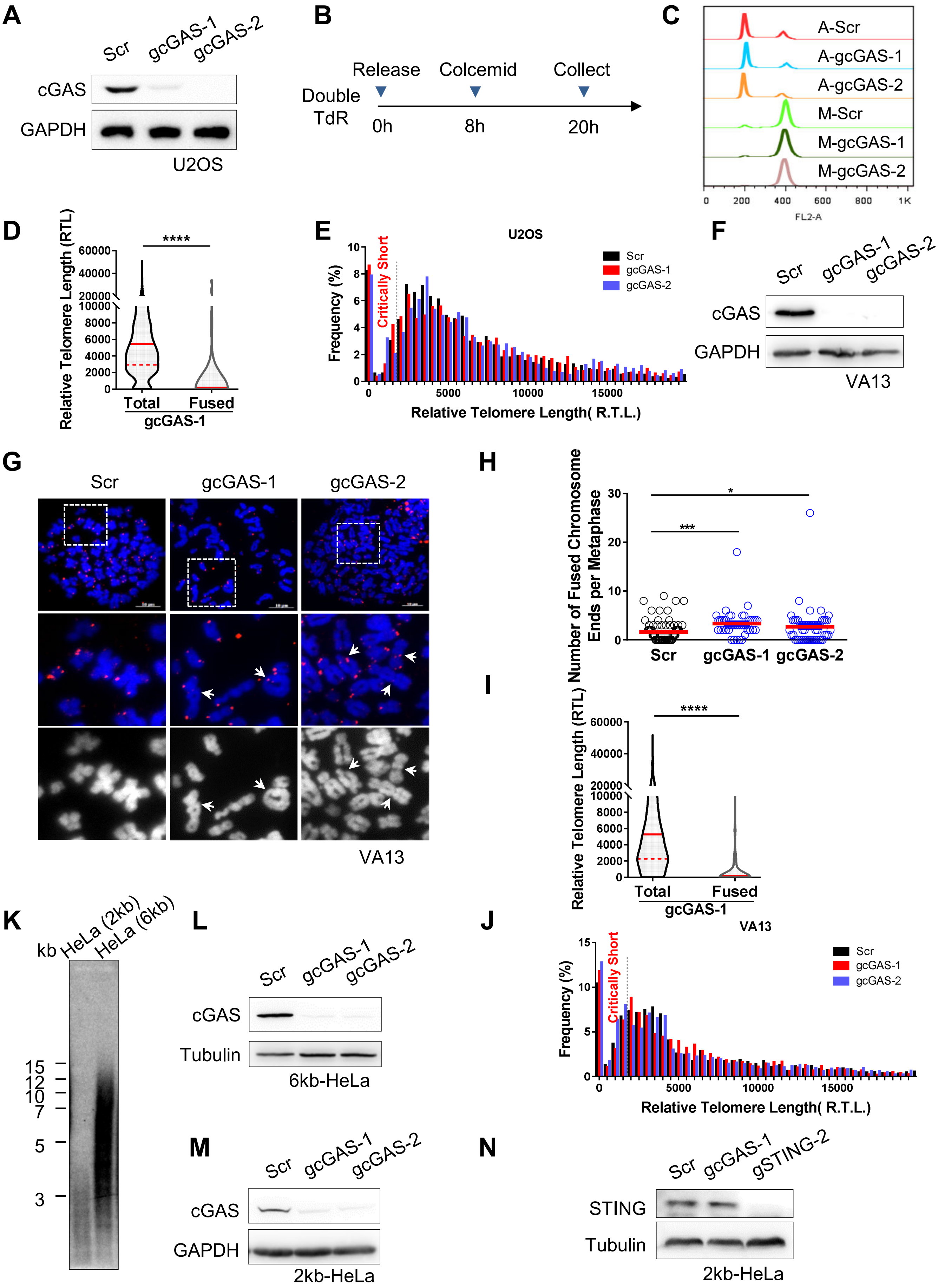

A
Scr
gcGAS-1
gcGAS-2
cGAS
GAPDH
U2OS
B
Release
Colcemid
Collect
Double TdR
0h
8h
20h
C
A-Scr
A-gcGAS-1
A-gcGAS-2
M-Scr
M-gcGAS-1
M-gcGAS-2
0 200 400 600 800 1K
FL2-A
D
Relative Telomere Length (RTL)
****
Total
Fused
gcGAS-1
E
U2OS
Frequency (%)
Critically Short
Scr
gcGAS-1
gcGAS-2
Relative Telomere Length(R.T.L.)
F
Scr
gcGAS-1
gcGAS-2
cGAS
GAPDH
VA13
G
Scr
gcGAS-1
gcGAS-2
VA13
H
Number of Fused Chromosome Ends per Metaphase
*
***
Scr
gcGAS-1
gcGAS-2
I
Relative Telomere Length (RTL)
****
Total
Fused
gcGAS-1
J
VA13
Frequency (%)
Critically Short
Scr
gcGAS-1
gcGAS-2
Relative Telomere Length(R.T.L.)
K
kb
HeLa (2kb)
HeLa (6kb)
15
12
10
7
5
3
L
Scr
gcGAS-1
gcGAS-2
cGAS
Tubulin
6kb-HeLa
M
Scr
gcGAS-1
gcGAS-2
cGAS
GAPDH
2kb-HeLa
N
Scr
gcGAS-1
gSTING-2
STING
Tubulin
2kb-HeLa

Figure S2.

(A) Immunoblot analysis cGAS in U2OS cells. cGAS was depleted by CRISPR/Cas9-based approach (gcGAS-1 or gcGAS-2, see method for details) and scramble sg-RNA (Scr) was used as a control.

(B) Time course of cell synchronization in C, G.

(C) Flow cytometry analysis of the cell cycle in asynchronous (A) or mitotic (M) control (Scr) and cGAS-deficient U2OS cells (gcGAS-1, gcGAS-2).

(D) Quantification of Figure **2A**. Relative lengths of fused telomeres were compared with that of total telomeres in cGAS-deficient U2OS cells (gcGAS-1).

(E) Quantification of Figure **2A**. The relative telomere length of mitotic control (Scr) and cGAS-deficient U2OS cells (gcGAS-1, gcGAS-2) determined by q-FISH.

(F) Immunoblot analysis cGAS in VA13 cells. The experiments were same as in A.

(G) FISH of telomeres on metaphase spreads to detect chromosome end-to-end fusions in VA13. The experiments were same as in Figure **2A**. Scale bars, 10 μm.

(H) Quantification of G. The number of fused chromosome ends per metaphase was given ($n \geq 44$ metaphase). All values are the average ±SEM of three independent experiments.

(I) Quantification of G. Relative lengths of fused telomeres were compared with that of total telomeres in cGAS-deficient VA13 cells (gcGAS-1).

(J) Quantification of G. The relative telomere length of mitotic control (Scr) and cGAS-deficient VA13 cells (gcGAS-1, gcGAS-2) determined by q-FISH.

(K) TRF assay detected telomeres length in 2kb, 6kb-HeLa cells.

(L) Immunoblot analysis cGAS in 6kb-HeLa cells. The experiments were same as in A.

(M) Immunoblot analysis cGAS in 2kb-HeLa cells. The experiments were same as in A.

(N) Immunoblot analysis STING in 2kb-HeLa cells. cGAS or STING was depleted by CRISPR/Cas9-based approach (gcGAS-1 or gSTING-2, see method for details) and scramble sg-RNA (Scr) was used as a control. The unpaired Student's two-tailed t-test was used to determine the statistical significance (*$P<0.05$, ***$P<0.001$, ****$P<0.0001$).

Figure S3

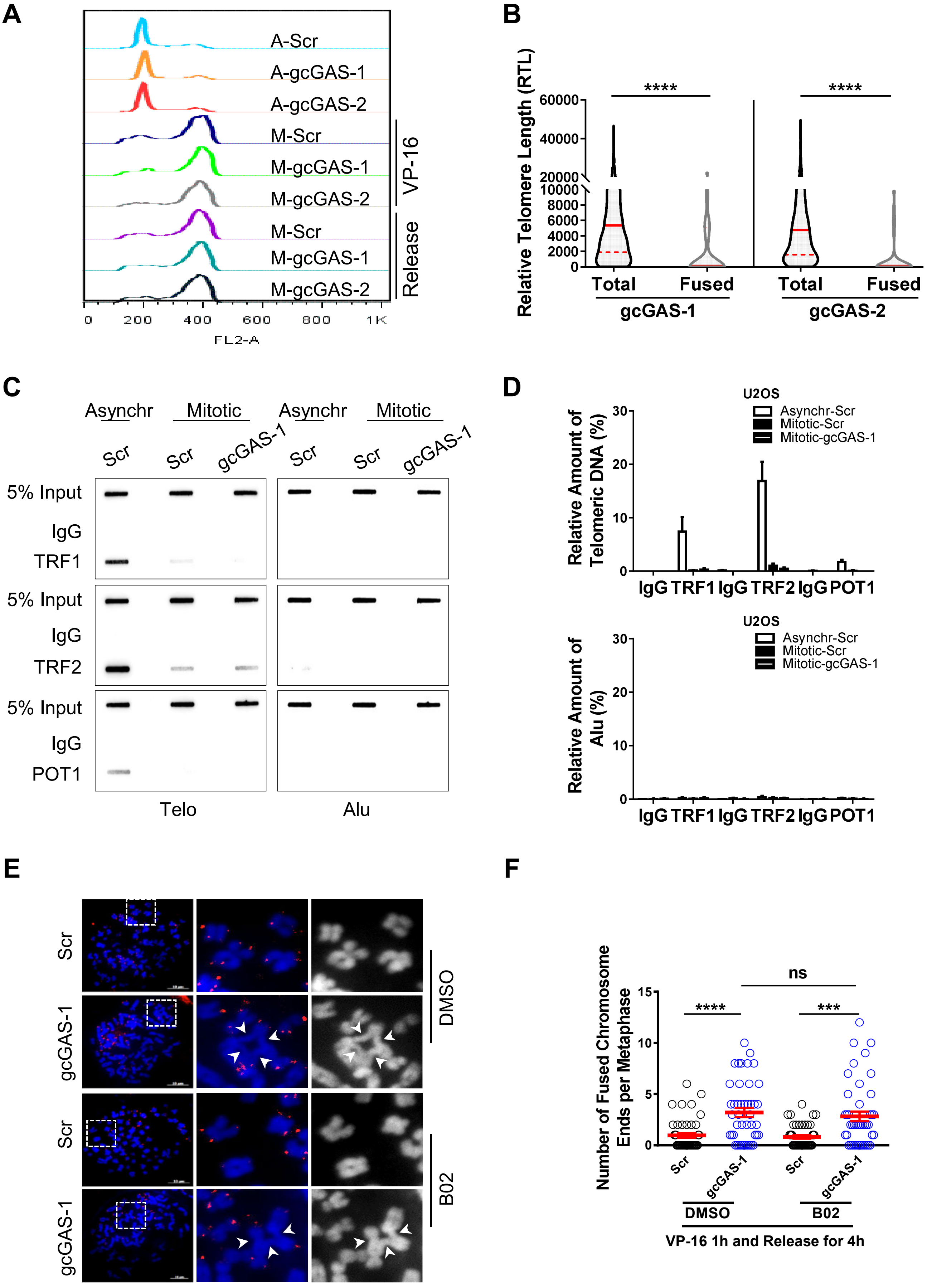
A
A-Scr
A-gcGAS-1
A-gcGAS-2
M-Scr
M-gcGAS-1
M-gcGAS-2
M-Scr
M-gcGAS-1
M-gcGAS-2
VP-16
Release
0
200
400
600
800
1K
FL2-A
B
Relative Telomere Length (RTL)
****
****
Total
Fused
Total
Fused
gcGAS-1
gcGAS-2
C
Asynchr
Mitotic
Asynchr
Mitotic
Scr
Scr
gcGAS-1
Scr
Scr
gcGAS-1
5% Input
IgG
TRF1
5% Input
IgG
TRF2
5% Input
IgG
POT1
Telo
Alu
D
U2OS
Asynchr-Scr
Mitotic-Scr
Mitotic-gcGAS-1
Relative Amount of Telomeric DNA (%)
IgG TRF1 IgG TRF2 IgG POT1
U2OS
Asynchr-Scr
Mitotic-Scr
Mitotic-gcGAS-1
Relative Amount of Alu (%)
IgG TRF1 IgG TRF2 IgG POT1
E
Scr
gcGAS-1
Scr
gcGAS-1
DMSO
B02
F
Number of Fused Chromosome Ends per Metaphase
ns
****
***
Scr
gcGAS-1
Scr
gcGAS-1
DMSO
B02
VP-16 1h and Release for 4h

Figure S3.

(A) Flow cytometry analysis of the cell cycle in asynchronous (A) or mitotic (M) 6kb-HeLa cells treated with VP-16. Control (Scr) or cGAS-depleted cells (gcGAS-1, gcGAS-2) were either harvested immediately following treatment (VP-16) or released for 4 h (Release) during mitosis.

(B) Quantification of Figure **3C**. Relative lengths of fused telomeres were compared with that of total telomeres in cGAS-deficient HeLa cells (gcGAS-1, gcGAS-2).

(C) ChIP analysis of TRF1, TRF2 and POT1 associating with telomeres in asynchronous (Asynchr) or mitotic U2OS cells. Control (Scr) and cGAS-deficient U2OS cells (gcGAS-1) were either asynchronous or synchronized at G1/S, released for 8 h and then treated with colcemid for 12 h. ChIP enriched DNA was used for slot blot and hybridization using telomeric G-rich probe or Alu probe.

(D) Quantification of C. The relative amount of enriched telomeric DNA was calculated (ChIP / Input, %). Alu was used as a control. All values are the average ±SEM of three independent experiments.

(E) FISH of telomeres on metaphase spreads to detect chromosome end-to-end fusions in VP-16 treated 6kb-HeLa cells in the presence of HR (B02) inhibitors. Control (Scr) or cGAS-depleted cells (gcGAS-1) were treated with VP-16 for 1 h, released for 4 h during mitosis, and then harvested for assay. Fusion events were indicated with arrows. Scale bars, 10 μm.

(F) Quantification of E. The number of fused chromosome ends per metaphase was given (n = 45 metaphase). All values are the average ±SEM of three independent experiments. The unpaired Student's two-tailed t-test was used to determine the statistical significance (***P<0.001, ****P<0.0001).

Figure S4

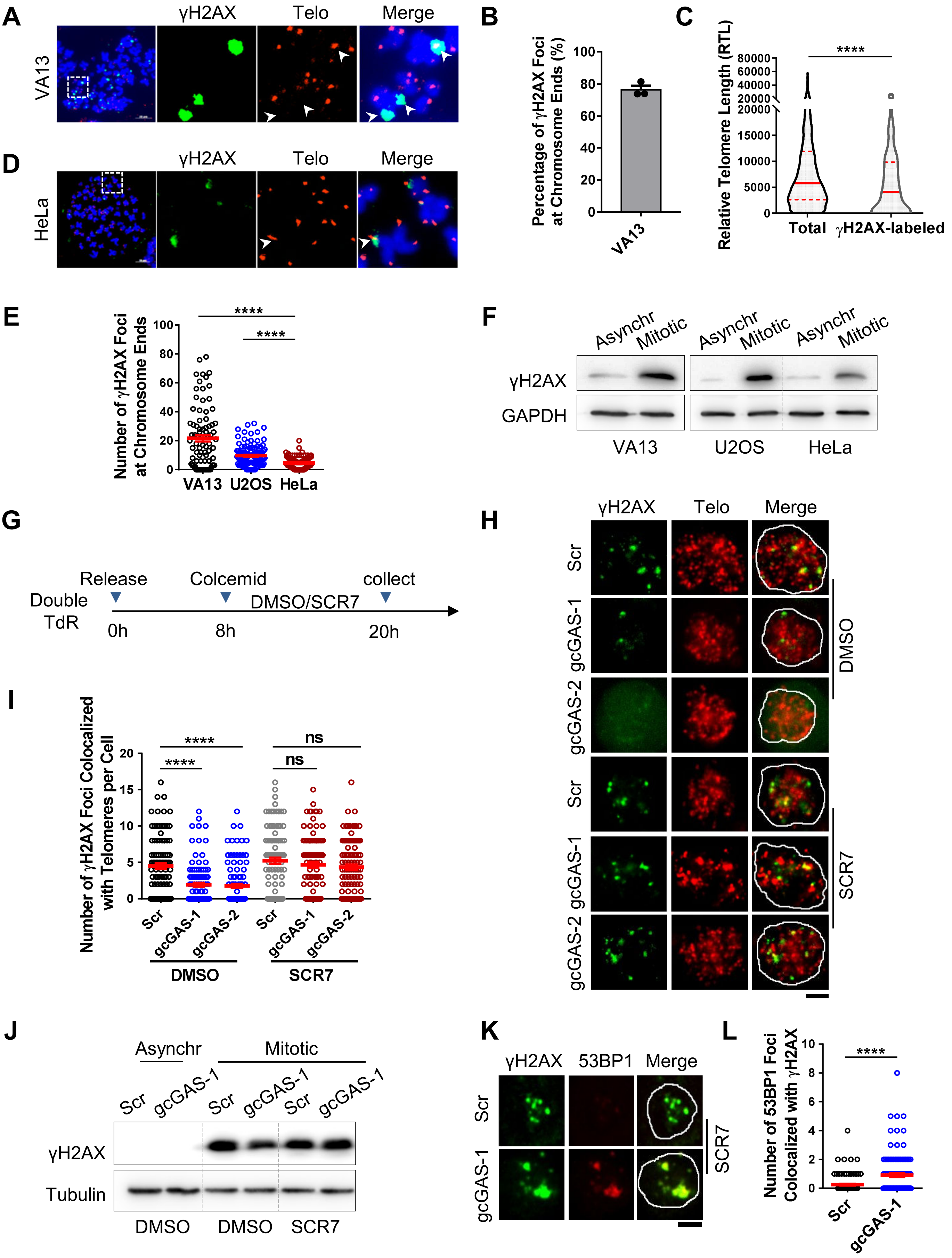

A
γH2AX
Telo
Merge
VA13
B
Percentage of γH2AX Foci at Chromosome Ends (%)
VA13
C
Relative Telomere Length (RTL)
****
Total
γH2AX-labeled
D
HeLa
E
Number of γH2AX Foci at Chromosome Ends
VA13
U2OS
HeLa
F
Asynchr
Mitotic
GAPDH
G
Double TdR
Release
Colcemid
collect
DMSO/SCR7
0h
8h
20h
H
Scr
gcGAS-1
gcGAS-2
DMSO
SCR7
I
Number of γH2AX Foci Colocalized with Telomeres per Cell
ns
J
Tubulin
K
53BP1
L
Number of 53BP1 Foci Colocalized with γH2AX

Figure S4.

(A) IF and FISH performed on metaphase spreads of VA13 cells to visualize γH2AX foci and telomeres, respectively. Mitotic TIF were indicated with arrows. Scale bars, 10 μm.

(B) Quantification of A. The percentage of γH2AX foci colocalizing with telomeres/chromosome ends was given. All values are the average ±SEM of three independent experiments.

(C) Quantification of A. Relative lengths of telomeres colocalized with γH2AX were compared with that of total telomeres.

(D) IF and FISH performed on metaphase spreads of HeLa cells to visualize γH2AX foci and telomeres, respectively. Mitotic TIF were indicated with arrows. Scale bars, 10 μm.

(E) Quantification of A, D and Figure **4A**. The number of γH2AX foci colocalized with telomeres/chromosome ends per metaphase between VA13, U2OS and HeLa was given.

(F) Immunoblot analysis γH2AX in asynchronous (Asynchr) and mitotic VA13, U2OS or HeLa cells.

(G) Time course of cell synchronization and SCR7 in H-L and Figure **4H-L**.

(H) IF-FISH analysis γH2AX and Telomeres in control (scr) and cGAS-deficient U2OS cells (gcGAS-1, gcGAS-2). Mitotic cells were cultured in the presence or absence of SCR7. Scale bars, 5 μm.

(I) Quantification of H. The number of γH2AX foci colocalized with telomeres per cell was given (n ≥ 100 cells).

(J) Immunoblot analysis of γH2AX in control (scr) and cGAS-deficient U2OS cells (gcGAS-1). Mitotic cells were cultured in the presence or absence of SCR7.

(K) IF detection of γH2AX and 53BP1 foci in control (scr) or cGAS-deficient U2OS cells (gcGAS-1). Mitotic cells were cultured in the presence of SCR7. Scale bars, 5 μm.

(L) Quantification of K. The number of 53BP1 colocalized with γH2AX foci per cell were calculated (n ≥ 100 cells). All values are the average ±SEM of three independent experiments. The unpaired Student's two-tailed t-test was used to determine the statistical significance (****P<0.0001).

Figure S5

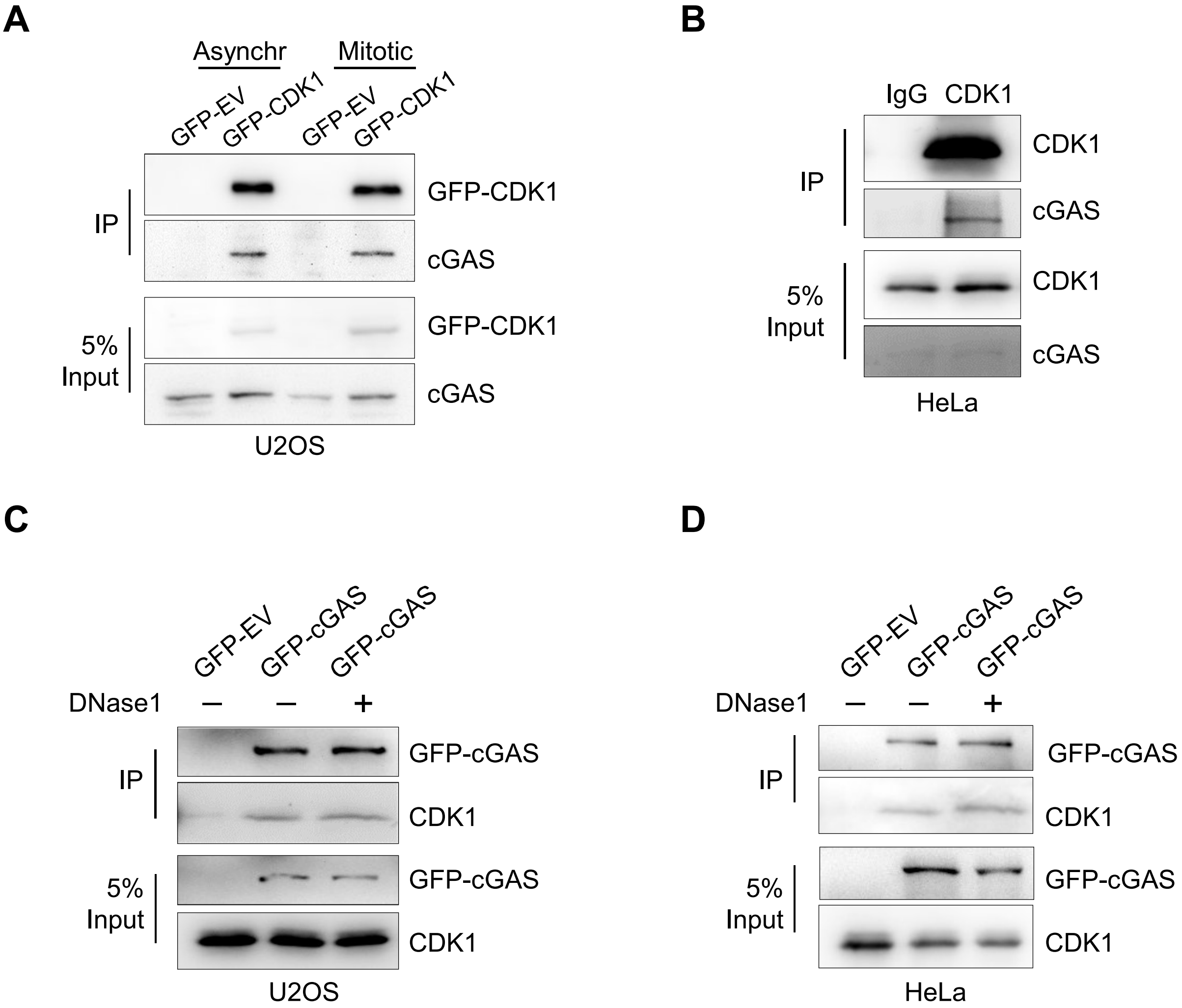

Figure S5.

(A) Co-IP assay using exogenously expressed GFP-CDK1 to determine the interaction of cGAS with CDK1 in asynchronous (Asynchr) or mitotic U2OS cells. GFP-EV was used as a negative control. Cells were either asynchronized or synchronized in mitosis as described above.

(B) Co-IP assay to determine the interaction of endogenous CDK1 with cGAS in mitotic HeLa cells. IgG was used as a negative control. Cells were synchronized in mitosis as described above.

(C) Co-IP assay using exogenously expressed GFP-cGAS to determine the interaction of CDK1 with cGAS in mitotic U2OS cells. Mitotic cell lysis was treated with DNase I prior to IP. Cells were synchronized in mitosis as described above.

(D) Co-IP assay using exogenously expressed GFP-cGAS to determine the interaction of CDK1 with cGAS in mitotic HeLa cells. Mitotic cell lysis was treated with DNase I prior to IP. Cells were synchronized in mitosis as described above.

Figure S6

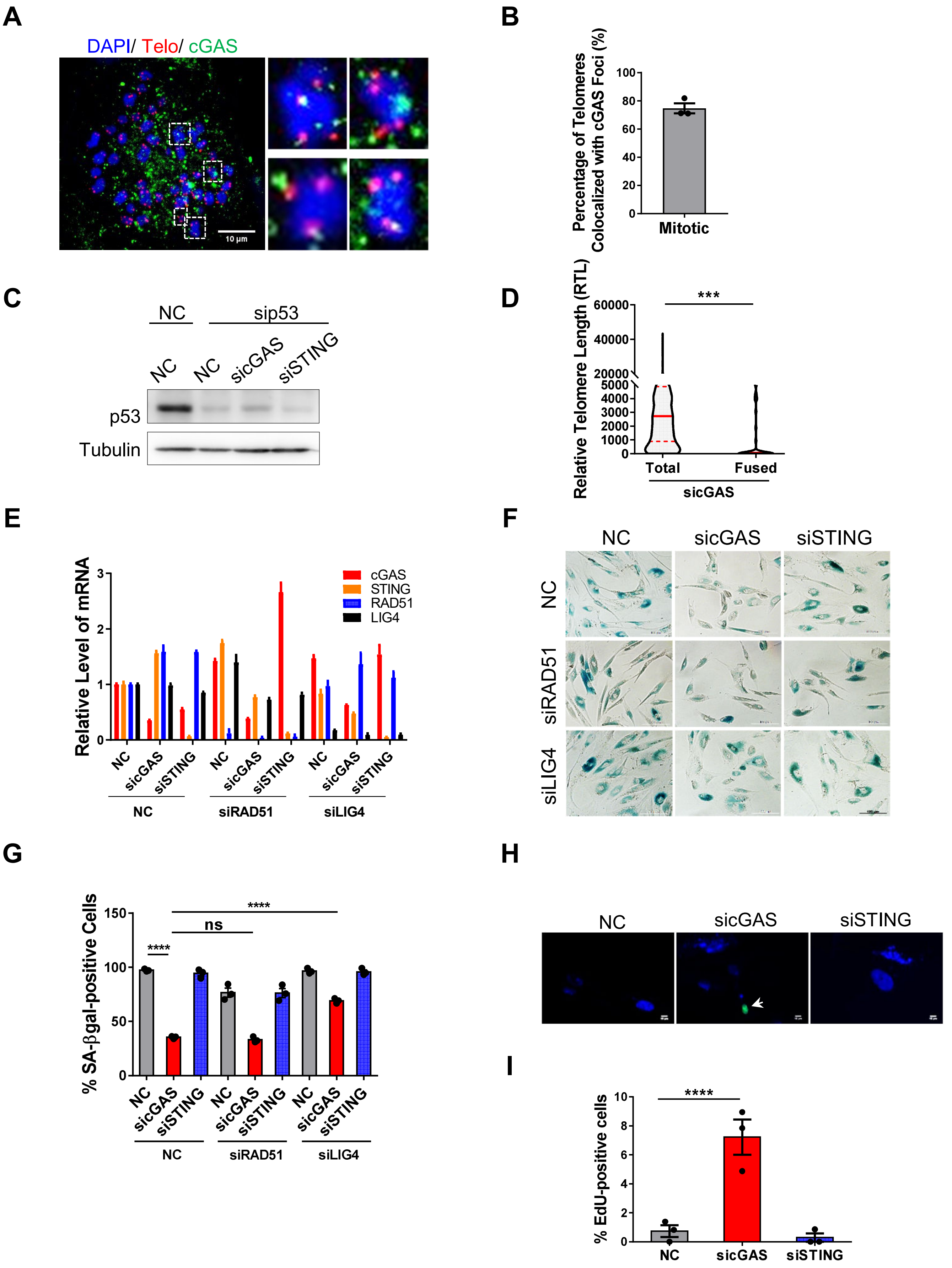
A
DAPI/ Telo/ cGAS
10 μm
B
Percentage of Telomeres Colocalized with cGAS Foci (%)
100
80
60
40
20
0
Mitotic
C
NC
sip53
NC
NC
sicGAS
siSTING
p53
Tubulin
D
Relative Telomere Length (RTL)
***
60000
40000
20000
5000
4000
3000
2000
1000
0
Total
Fused
sicGAS
E
Relative Level of mRNA
cGAS
STING
RAD51
LIG4
NC
sicGAS
siSTING
NC
siRAD51
siLIG4
F
NC
sicGAS
siSTING
NC
siRAD51
siLIG4
G
% SA-βgal-positive Cells
150
100
50
0
****
ns
****
NC
sicGAS
siSTING
NC
siRAD51
siLIG4
H
NC
sicGAS
siSTING
I
% EdU-positive cells
10
8
6
4
2
0
****
NC
sicGAS
siSTING

Figure S6.

(A) Visualization of endogenous cGAS and telomeres in mitotic BJ cells. Cells were treated with colcemid for 20 h and then subjected to metaphase spread followed by IF/FISH. Scale bars, 10 μm.

(B) Quantification of A. Percentage of telomeres colocalizing with cGAS foci was calculated. All values are the average ±SEM of three independent experiments.

(C) Immunoblot analysis of p53 in pre-senescent BJ fibroblast cells sequentially transfected with si-scramble (NC) or sicGAS or siSTING for 12 days and then with sip53 for 4 days.

(D) Quantification of Figure 6G. Relative lengths of fused telomeres were compared with that of total telomeres in cGAS and p53-double knocked down BJ fibroblast cells.

(E) Detection of mRNA levels of cGAS, STING, RAD51, and LIG4 in BJ cells transfected with NC or sicGAS or siSTING in combination with transfection of siRAD51 or siLIG4 as indicated. mRNA levels were quantified by qPCR.

(F) SA-β-Gal staining of pre-senescent BJ fibroblast cells transfected with si-scramble (NC) or sicGAS or siSTING in combination with transfection of siRAD51 or siLIG4 as indicated. After transfection, cells were cultured for 12 days and subjected to SA-β-Gal staining. Scale bars, 100 μm.

(G) Quantification of F. The percentage of SA-β-Gal positive cells was calculated ($n \geq 100$ cells). All values are the average ±SEM of three independent experiments.

(H) EdU labeling was used to indicate S phase BJ fibroblast cells. Pre-senescent BJ fibroblast cells sequentially transfected with si-scramble (NC) or sicGAS or siSTING for 24 days. Corresponding siRNA was transfected every 4 days. EdU-labeled cell was indicated with arrow. Scale bars, 10 μm.

(I) Quantification of H. The percentage of cells with EdU was determined ($n \geq 100$ cells). All values are the average ±SEM of three independent experiments. The unpaired Student's two-tailed t-test was used to determine the statistical significance (***$P<0.001$, ****$P<0.0001$).
